# Introduction to “Chemical biology of metals”

**DOI:** 10.1039/d4cb90017k

**Published:** 2024-05-10

**Authors:** Angela Casini, Hui Chao, Hongzhe Sun, Christopher J. Chang

**Affiliations:** a Chair of Medicinal and Bioinorganic Chemistry, School of Natural Sciences, Department of Chemistry, Technical University of Munich Garching Germany; b MOE Key Laboratory of Bioinorganic and Synthetic Chemistry, State Key Laboratory of Anti-Infective Drug Discovery and Development, Guangdong Basic Research Center of Excellence for Functional Molecular Engineering, School of Chemistry, Sun Yat-Sen University Guangzhou 510006 P. R. China; c Department of Chemistry, State Key Laboratory of Synthetic Chemistry, CAS-HKU Joint Laboratory of Metallomics on Health and Environment, The University of Hong Kong Pok Fu Lam Hong Kong S.A.R. P. R. China; d Department of Chemistry and Molecular and Cell Biology, University of California Berkeley Berkeley California USA chrischang@berkeley.edu

## Abstract

Angela Casini (Technical University of Munich, Germany), Hui Chao (Sun Yat-Sen University, China), Hongzhe Sun (University of Hong Kong, China), and Christopher J. Chang (University of California, Berkeley, United States) introduce the themed collection on ‘Chemical biology of metals’.
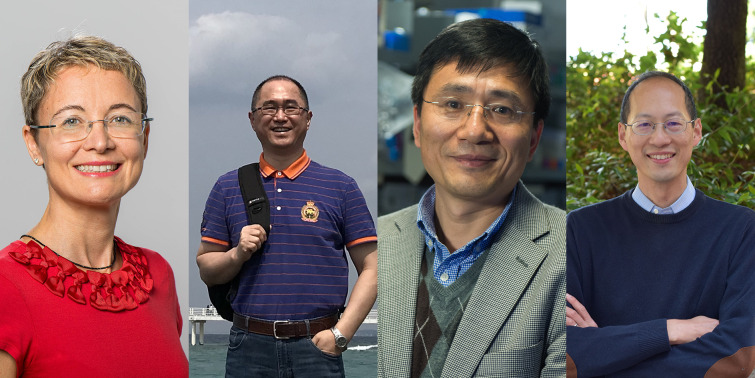

This themed collection highlights articles showcasing the ongoing interest in the chemical biology of metals. Recent years have witnessed significant advances in the development of new imaging probes that can probe the physiological and pathological roles of metals in biology and/or leverage unique properties of metal coordination chemistry and metal-based medicines. In addition, significant progress has been made in understanding metallo-medicines and studying metal-based catalysis and signalling in living systems. We therefore have collected contributions that are representative of the tremendous promise and pace of growth in this area.

Metal-based imaging, including theranostic approaches, is a frontier area in the chemical biology of metals. Abergel's group develops a siderocalin-antibody targeting system for theranostic approaches, where they innovatively use this scaffold to bind dual ^86^Y/^90^Y siderophores for potential precision medicine applications (https://doi.org/10.1039/D3CB00050H). Holland's group reports a novel approach to prostate-specific antigen detection as a potential cancer diagnostic based on Designed Ankyrin Repeat Proteins (DARPins) bearing a gallium radio-label (https://doi.org/10.1039/D3CB00010A). Boros and Cotruvo present a first-generation study exploring the use of lanmodulin (LanM) as a novel scaffold for medicinally relevant radioisotopes: ^177^Lu, ^132/135^La and ^89^Zr, setting the foundation for the broader use of these naturally-occurring f-element chelates in radiochemistry (https://doi.org/10.1039/D3CB00020F).

Also, as part of this collection, several studies focus on metal-based therapies, with many expanding the scope of metallo-medicines beyond traditional platinum anticancer drugs. Heidary and Glazer and co-workers develop a structurally-distinct ruthenium-based counterpart to the widely-used phenanthriplatin monofunctional reagent for cancer therapy and show that it induces cell death *via* ribosome biogenetic stress (https://doi.org/10.1039/D2CB00247G). Wilson and colleagues describe nitride-bridged osmium medicines with greater stability compared to their oxo counterparts, which address a metal-based target, the calcium uniporter, for protection against neuronal damage (https://doi.org/10.1039/D2CB00189F). Awuah and colleagues describe a three-coordinate gold N-heterocyclic carbene (NHC) complex for anticancer treatment, providing a robust chemical scaffold targeting mitochondrial function for the treatment of glioblastoma as a lethal cancer (https://doi.org/10.1039/D3CB00051F). Xia and Sun and their teams report an interesting dual-action approach to antibiotic treatment, where a gallium-flavonoid can act as a bactericidal reagent by interfering with both native iron metabolism and quorum sensing (https://doi.org/10.1039/D3CB00033H). Dreab and Bayse explore how metalation of the N-terminal amino terminal copper and nickel (ATCUN) binding motif of piscidins may enhance their antimicrobial properties, with a critical feature being to balance electrostatics to augment membrane association (https://doi.org/10.1039/D3CB00035D).

Advances in platinum medicines also continue to be an active area. Guo, He, and Chen and their co-workers develop an impactful application of aggregation-induced emission (AIE) for photodynamic therapy, using platinum as a heavy-metal center to increase singlet oxygen production (https://doi.org/10.1039/D3CB00113J). Zhu's team reviews the state-of-the-art in organelle-targeting platinum anticancer drugs, where diverse approaches presage the potential of this direction in limiting side effects and drug resistance (https://doi.org/10.1039/D3CB00087G).

Metallomics and metalloproteomics, from the identification of new metal–protein and metal–metabolite interactions at the systemic level and targets of metal-based medicines, is an area of rapid growth for the field. Heffern and her team identify a pair of fascinating metal–hormone interactions by showing that both copper and zinc bind oxytocin and can amplify downstream MAP kinase signalling (https://doi.org/10.1039/D2CB00225F). Wang's laboratory presents a study revealing global protein targets of the classic anticancer metallodrug cisplatin, where using cysteine activity-based protein profiling (ABPP) they identify methionine aminopeptidases 1 (MetAP1) as a novel potential target for improving cytotoxicity of cisplatin to avoid tumor resistance (https://doi.org/10.1039/D3CB00042G). DeRose's laboratory reports a novel set of chemical tools to help better understand platinum-induced nucleolar stress, using a systematic series of compounds to elucidate structure–activity relationships to enable incorporation of azide enrichment handles for click chemistry, while maintaining compound efficacy (https://doi.org/10.1039/D3CB00055A). Codd and colleagues present a smart reduction-cleavage bait approach to identify new targets of siderophore-mediated iron uptake, revealing nickel superoxide dismutase's involvement in desferrioxamine B and a novel metal–metal crosstalk for further study (https://doi.org/10.1039/D3CB00097D). Michel's laboratory uses a large-scale global profiling approach to identify zinc finger protein sites as major targets of persulfidation, adding to the growing body of work in metal-redox biology and showing that sulfur-based post-translational modification is a widespread regulation of one of the most common metal-binding motifs in the genome and proteome (https://doi.org/10.1039/D3CB00106G).

Finally, two reviews highlight one of the most exciting and important areas for the chemical biology of metals: their broad contributions to neuroscience. Yoo, Han, and Lim present a timely review of metals in neuroscience. They focus on advances in identifying and understanding the action of metals in binding neurotransmitters to tune their function, including oxidation, polymerization, and aggregation (https://doi.org/10.1039/D3CB00052D). Quintanar and her co-workers write an interesting opinion piece that brings together disparate metal-dependent disease vulnerabilities through the viewpoint of metal-induced protein aggregation, linking neurodegenerative Alzheimer's, cataracts, and diabetes through a common metal-centric analysis (https://doi.org/10.1039/D3CB00145H).

Taken together, this collection showcases the breadth and depth of metals in chemical biology with contributions from all over the world. We hope that readers will be inspired by these representative studies to join us in exploring the numerous opportunities to study the elements of life and translate this fundamental knowledge to better understand, and subsequently diagnose, and treat diseases.

## Supplementary Material

